# Synthesis of graft polyrotaxane by simultaneous capping of backbone and grafting from rings of pseudo-polyrotaxane

**DOI:** 10.3762/bjoc.10.269

**Published:** 2014-11-04

**Authors:** Kazuaki Kato, Katsunari Inoue, Masabumi Kudo, Kohzo Ito

**Affiliations:** 1 Department of Advanced Materials Science, Graduate School of Frontier Sciences, The University of Tokyo, 5-1-5 Kashiwanoha, Kashiwa, Chiba 277-8561, Japan; 2 Advanced Softmaterials Inc., Tokatsu Techno Plaza 403, 5-4-6 Kashiwanoha, Kashiwa, Chiba, 277-0882, Japan

**Keywords:** branch structure, cyclodextrin, end capping, graft polyrotaxane, polyrotaxane

## Abstract

Graft polyrotaxanes, with poly(ε-caprolactone) (PCL) graft chains on the ring components were synthesized by the simultaneous ring-opening polymerization of ε-caprolactone from both ends of the backbone polymer, an end-functionalized polyethylene glycol (PEG) and the formation of inclusion complexes with α-cyclodextrin (α-CD). PEG with multiple functional groups at each end was prepared by the condensation of PEG-amine and D-gluconic acid; the PEG derivative formed an inclusion complex with α-CD. The polymerization of multiple hydroxy groups at the backbone ends resulted in a star-shaped end group, which served as a bulky capping group to prevent dethreading. In contrast, PEG with only one hydroxy group at each end did not produce polyrotaxanes, indicating that single PCL chains were too thin to confine α-CDs to the complex. In addition, the grafting polymerization proceeded properly only when robust hydrogen bonds formed between α-CDs were dissociated using a basic catalyst. Since the dissociation also induced dethreading, kinetic control of the polymerization and dissociation were crucial for producing graft polyrotaxanes. Consequently, this three-step reaction yielded graft polyrotaxanes in a good yield, demonstrating a significant simplification of the synthesis of graft polyrotaxanes.

## Introduction

Polyrotaxanes, interlocked molecules composed of polymer chains and cyclic molecules, are characterized by intramolecular mobility, namely rotation and sliding of the rings along the chains. Cyclodextrins (CDs) are often used as the cyclic components owing to their versatile and efficient inclusion abilities toward polymer guests [1–2]. The advantage of CD usage arises from the multiple hydrogen bonds formed when CDs are threaded onto polymer chains. The threaded CDs are oriented face-to-face or tail-to-tail in the direction of the backbone chain, so that their hydroxy groups can efficiently form hydrogen bonds with those of neighboring CDs [3–4]. At the same time, however, hydrogen bonds prevent the characteristic mobility of polyrotaxanes. The hydrogen-bonded CDs form a robust columnar crystal, and thus the polyrotaxanes become insoluble in most solvents [1]. Therefore, specific solvents that prevent hydrogen bond formation, such as DMSO and DMAc/Li, are used to dissolve polyrotaxanes and to enable the intramolecular mobility [5–6]. For the same reason, chemical modifications of the CD groups drastically increase the solubility [7].

Modifications of CDs with polymer chains enable the characteristic mobility even without the use of solvents. Graft polyrotaxanes (GPRs), polyrotaxanes with grafted chains on CDs, have been synthesized by “grafting to” [8–10] and “grafting from” [11–12] methods with various polymers. GPRs with PMMA grafts exhibit repulsion between the CDs to stretch the backbone polymer, resulting in the shear-induced decomposition of the rotaxane structures [11]. GPRs with poly(ε-caprolactone) (PCL) as a flexible graft chain, which exhibits a much lower glass transition temperature than PMMA, have been cross-linked to yield flexible elastomers [11]. Such elastomers exhibit an excellent scratch resistance, and thus can be employed as a commercial coating material [13].

**Scheme 1:**
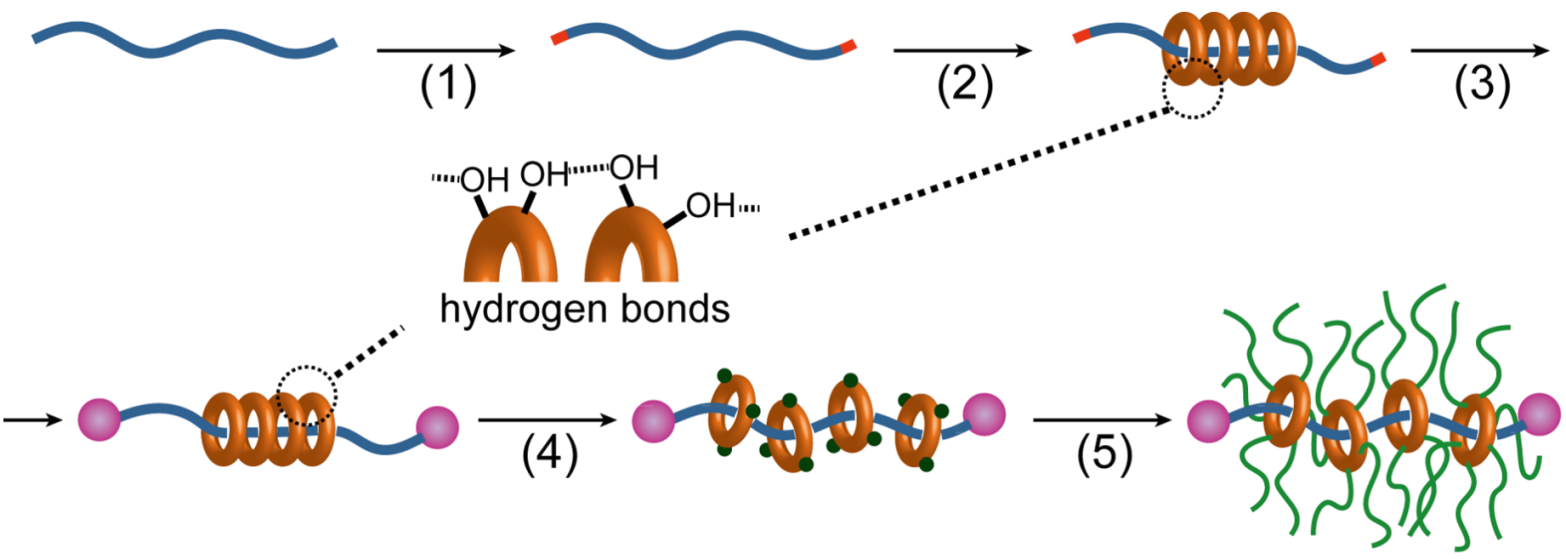
General synthetic scheme of graft polyrotaxane (GPR) consisting of 1) functionalization of polymer ends, 2) complexation, 3) end capping, 4) chemical modification of rings, and 5) grafting.

Although such GPRs are valuable both scientifically and industrially, their syntheses are very complicated. A typical synthesis consists of the following five steps, as shown in Scheme 1: 1) functionalization of both ends of the polymer chains, 2) formation of inclusion complex with CDs, 3) end capping, 4) chemical modification of CDs, and 5) grafting. The first three steps constitute the synthesis of polyrotaxane. The fourth process is necessary to dissociate the columnar crystal of CDs formed by hydrogen bonds and to disperse the CDs within the backbone; without this step the grafting reaction hardly occurs or produces insoluble products [10,14]. When the modification process is carried out prior to the second process and the modified CDs are used for the complexation, an inclusion complex is not obtained because the hydrogen bonds, the main driving force for complexation, are not formed [3,15–16]. The end-capping process should be carried out before chemical modification; otherwise the modification will induce the dissociation of the inclusion complex. Therefore, although the crystal formation (driven by the hydrogen bonds of the CDs) is necessary for complexation, it complicates the synthesis of GPRs.

**Scheme 2:**
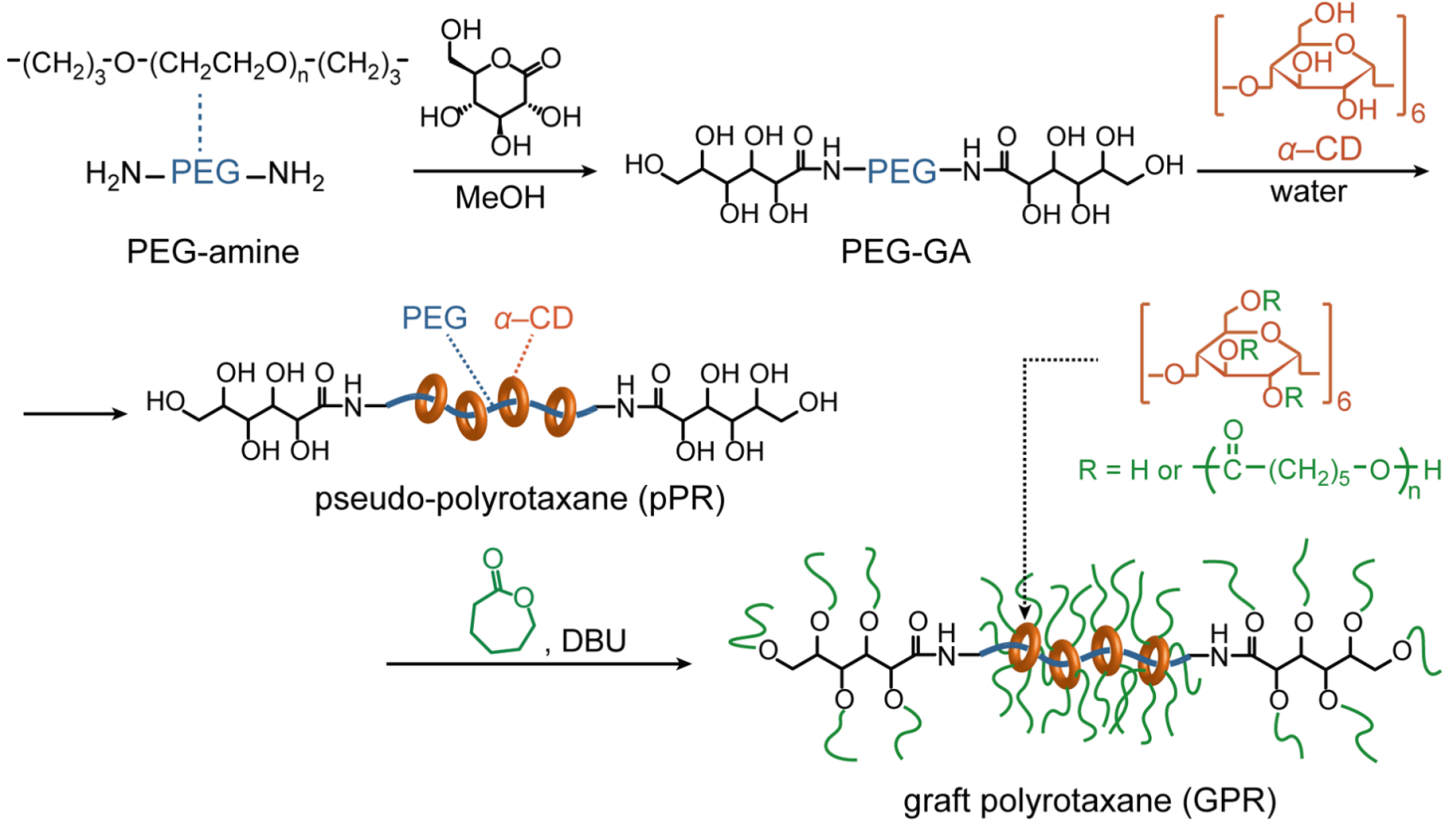
Simplified synthetic scheme of graft polyrotaxane (GPR).

Here, we propose a new synthetic scheme for GPRs based on simultaneous end-capping and grafting reactions to directly produce a pseudo-polyrotaxane (pPR), as shown in Scheme 2. Since the CDs and the backbone polymer ends have hydroxy groups, the ring-opening polymerization of ε-caprolactone can be initiated from the CDs or the backbone ends. PCL chains grafted from the polymer ends may behave as capping groups when more than two chains are grafted to make branched polymer ends, as the chain itself is not bulky enough. Notably, PCL is thin enough to form an inclusion complex with α-CD, the smallest CD [17]. In addition, DBU was employed not only as a polymerization catalyst but also as a base to prevent hydrogen bond formation between CDs, so that the ring components would disperse on the backbone. However, this can also induce the dethreading of the CDs. Since the end capping, modification of CDs, and grafting were integrated, the synthesis of GPR was much more facile.

## Results and Discussion

Multifunctionalization of the polymer ends is necessary for the synthesis of GPR, as shown in Scheme 2, because the PCL chain is thin enough to form an inclusion complex with α-CD, indicating that the PCL chains at the ends of PEG cannot prevent the dethreading of the CDs. We demonstrated the complexation between α-CD and PCL-PEG-PCL triblock copolymers. The triblock copolymer was synthesized by the ring-opening polymerization of ε-caprolactone from the same PEG-amine (*M*_n_ = 18,400), and each PCL block had *M*_n_ of ca. 3,000. When an aqueous solution of the copolymer is mixed with that of α-CD, the solution gradually became turbid until the white precipitate of pseudo-polyrotaxane (pPR) was obtained. Accordingly, the grafting of a single chain of PCL at the end of the backbone polymer was unlikely to prevent dethreading. However, star-shaped PCL polymers were expected to be sufficiently bulky stoppers.

**Figure 1:**
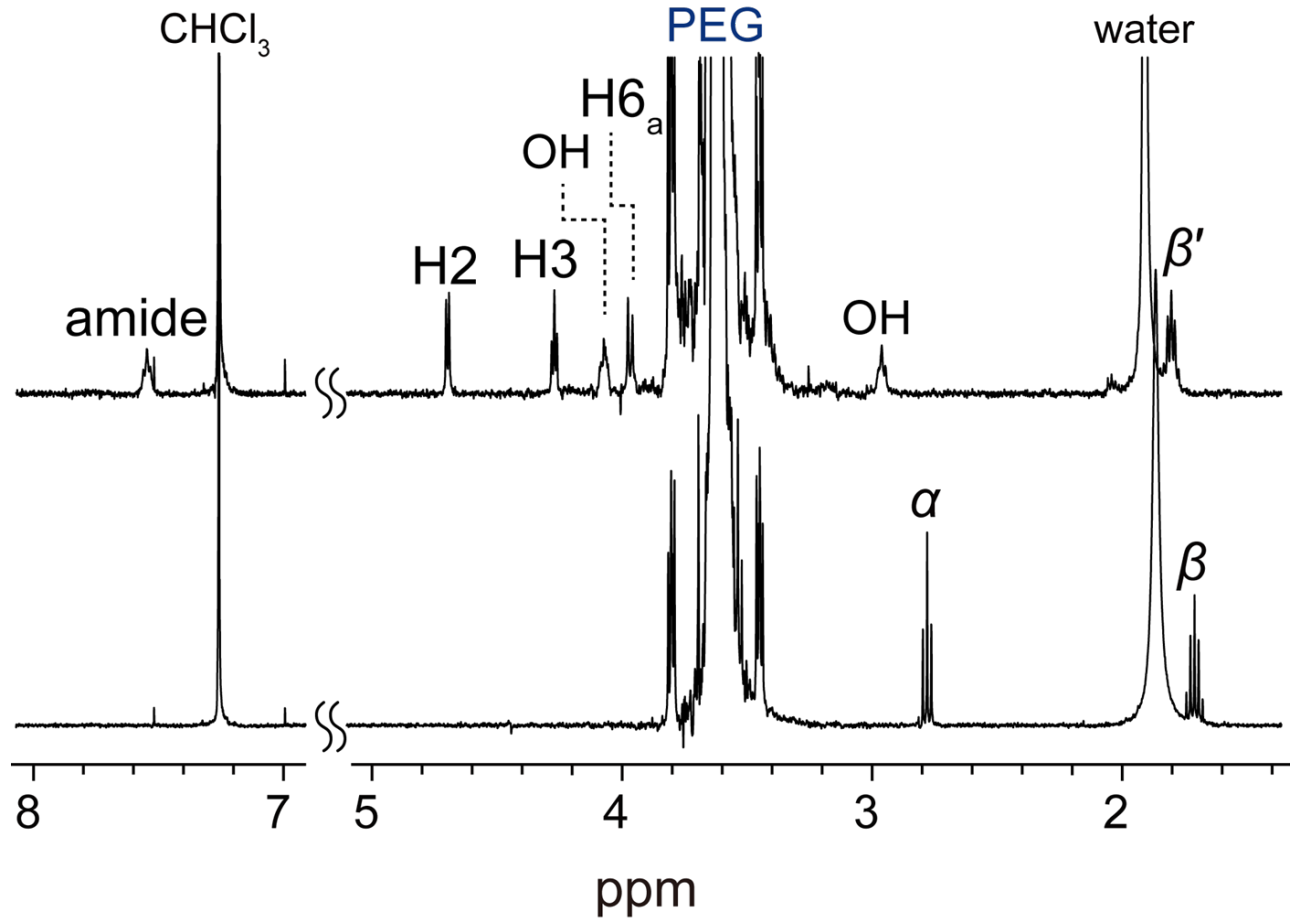
Partial ^1^H NMR (400 MHz, CDCl_3_, 298 K) spectra of PEG-GA (above) and PGE-amine (below).

Multifunctionalized PEG was readily obtained by the reaction between PEG-amine and D-gluconic acid. The ^1^H NMR spectrum of PEG-GA is shown in Figure 1. Although some protons of PEG-GA overlapped with the main peak of the repeating unit around 3.6 ppm, other protons were assigned as indicated in Figure 1. The shifts attributed to the α- and β*-*methylene of the amino group indicated the quantitative conversion of the end groups. The FTIR spectrum also indicated the formation of an amide bond, as noted by the amide I and II vibrations at 1649 and 1537 cm^−1^, respectively. The SEC chromatogram revealed that the *M*_n_ and *M*_w_ values of PEG-GA were 20,600 and 23,700, respectively, confirming the lack of degradation in the polymer chain. The obtained PEG-GA was dissolved in water and mixed with an aqueous solution of α-CD to form the inclusion complex. The initially clear solution turned into a turbid gel, indicating the formation of pPR. Although the multifunctionalized ends of PEG-GA were larger than those of PEG-amine, they seemed to be small enough to thread the cavity of α-CD.

Simultaneous grafting of PCL from both α-CDs and the multifunctionalized ends of the pPR backbone yielded GPRs depending on the reaction conditions. The ring-opening polymerization of ε-caprolactone initiated by pPR began under heterogeneous conditions. As the polymerization progressed, the reaction mixtures became homogeneous, viscous liquids. The conditions for the ring opening polymerization are shown in Table 1. The polymerization of ε-caprolactone did not occur at 0 °C and remained heterogeneous; thus, unmodified, dethreaded α-CD was detected by SEC in a suitable solvent for α-CD (run 5). At 130 °C, the reaction mixture became homogeneous within several minutes, indicating the rapid polymerization of ε-caprolactone. Although the polymerization proceeded from α-CD, the grafted α-CDs were not threaded by the backbone polymer, as confirmed by the SEC peak corresponding to *M*_n_ = 11,000 and *M*_w_ = 15,000 (run 4). These results suggested accelerated dethreading at high temperatures, although it is unknown whether dethreading occurred before or after the grafting reaction. The polymerization was initiated by DBU, a strong base, which deprotonated the hydroxy groups. Therefore, the hydrogen bond formation between the CDs of pPR did not form. As a result, the robust columnar crystals in pPR were dissociated. This mechanism was also supported by the fact that polyrotaxanes composed of PEG and α-CD are only soluble in water in the presence of a base [18]. Notably, the direct grafting from polyrotaxane with tin catalysts rather than DBU yielded a cross-linked GPR as a gel-like insoluble solid, indicating the necessity of the dissociation of the columnar crystal. Accordingly, GPR is not obtained if the polymerization from the ends of the backbone is slower than the dethreading initiated by the base catalyst, which is necessary for grafting.

**Table 1:** Conditions and results of ring-opening polymerization from pPR.

	polymerization conditions		GPR	
run		*M*_n_	*M*_w_	Yield^a^

1	60 °C, 48 h	97,000	115,000	69%
2	rt, 6 h → 40 °C, 24 h^b^	181,000	209,000	33%
3	rt, 48 h	167,000	197,000	3%
4	130 °C, 10 h	(11,000)^c^	(15,000)^c^	0%
5	0 °C, 7 h	(780)^c^	(850)^c^	0%

^a^Yields based on polymer backbone. ^b^Temperature increased after 6 h. ^c^Molecular weights of byproducts.

**Figure 2:**
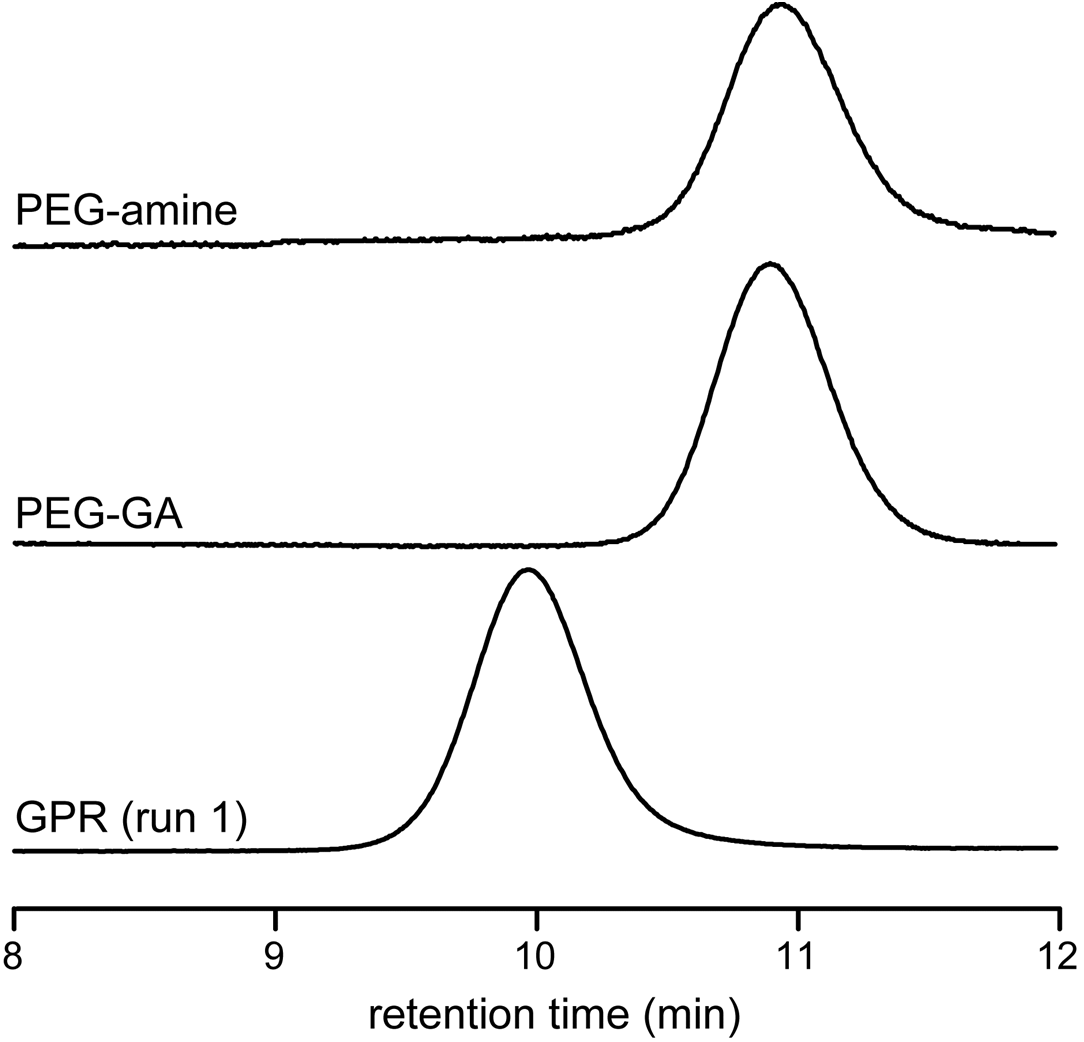
SEC traces of GPR (run 1 in Table 1) and PEG derivatives. Eluents: DMSO/LiBr; detection: differential refractive index.

**Figure 3:**
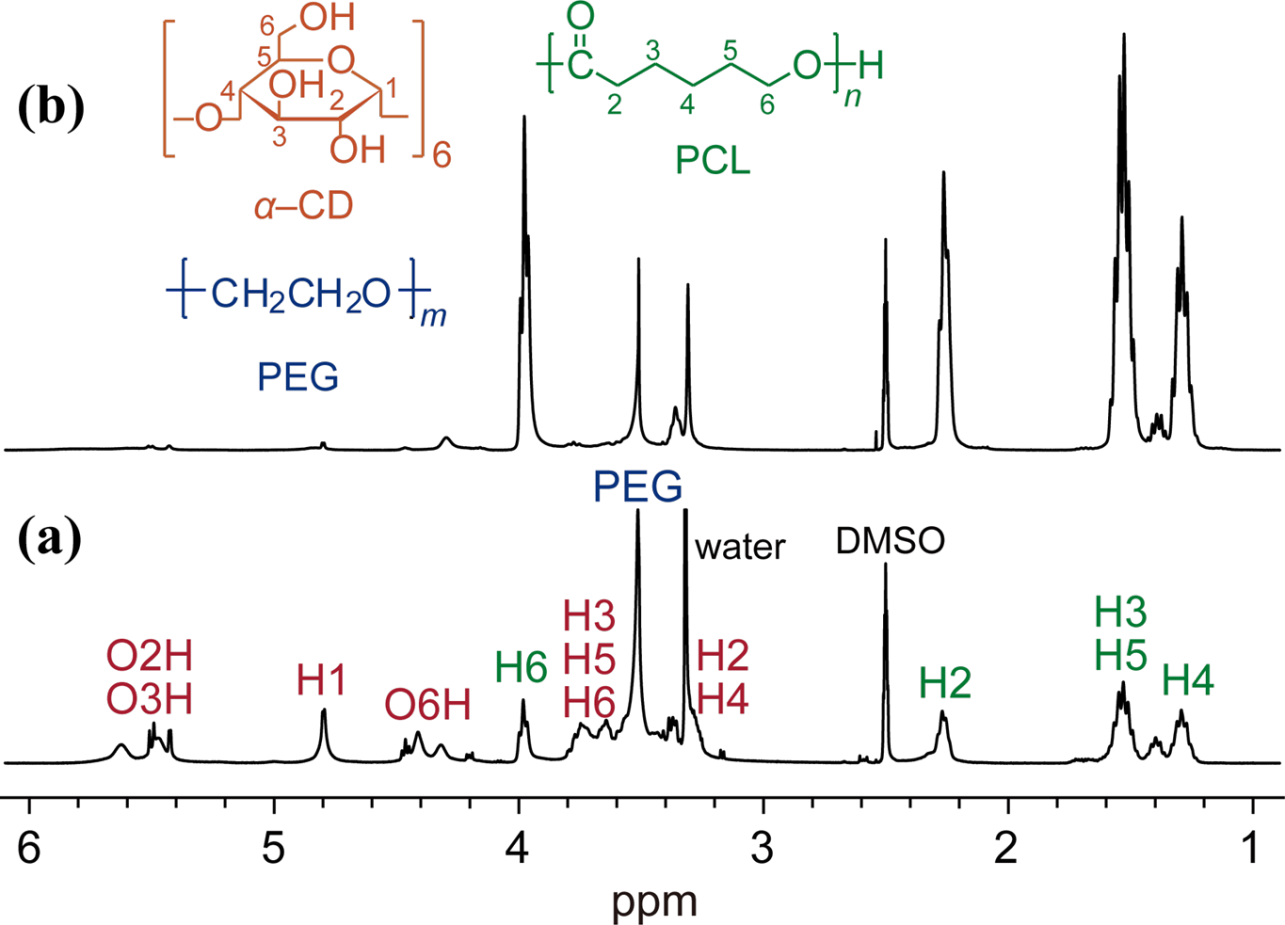
^1^H NMR spectra (400 MHz, DMSO-*d*_6_, 298 K) of GPRs: (a) run 1 and (b) run 2.

The dethreading was decelerated at decreased temperatures. The reaction carried out at 60 °C yielded a high molecular weight product (run 1). The chromatogram of the product is shown with that of the PEG derivatives in Figure 2. This result indicated that the product had a higher molecular weight than PEG-GA. Notably, the product exhibited a low polydispersity index, *M*_w_/*M*_n_ = 1.19, which was only slightly larger than that of PEG-GA. The significant increase in the molecular weight suggested the production of GPR, which was also supported by the ^1^H NMR spectrum. Figure 3a shows the ^1^H NMR spectrum of the product. The spectrum consisted of three components, namely PEG, α-CD, and PCL, and the peaks were assigned as shown in the figure. The coverage, which is a measure of the CD packing density along the backbone polymer, was roughly estimated to be 60%, though many signals of α-CD and water overlapped. The average molecular weight of PCL was estimated from the spectrum to be ca. 400 based on the assumption that each α-CD had a single graft chain. This result suggested that the PCL chains on the α-CDs of GPR were short and sparse, indicating that the polymerization of ε-caprolactone did not proceed effectively. However, the crude GPR exhibited the presence of a byproduct observed around 11.6 min, which was grafted α-CD with a molecular weight of ca. 7,000, which corresponded to *M*_W_ of ca. 6,000 of PCL chains based on the same assumption. The difference in the molecular weights of PCL may imply that the polymerization proceeded from free α-CDs prior to that of α-CDs in GPR. Indeed, the polymerization hardly occurred from the α-CDs of the polyrotaxanes, because the hydrogen bonds between the rings that form robust columnar crystals drastically reduced the accessibility of the monomers [11,14]. In addition, α-CD itself works as a catalyst in the polymerization of ε-caprolactone [19]. Thus, the polymerization of ε-caprolactone owing to the α-CDs should be much faster than that owing to α-CDs in GPR. Since many of the monomers were consumed by the free α-CDs, the degree of polymerization of the PCL chains in GPR remained low. On the other hand, the polymerization from the end groups of the backbone sufficiently proceeded to prevent the dethreading, because the yield of GPR was high. The polymerization from the hydroxy groups of the backbone ends could be faster than that from α-CDs, because the end groups would not form strong hydrogen bonds to inhibit the polymerization.

The degree of polymerization of PCL from the α-CDs in GPR increased when the reaction temperature decreased (run 3), even though the reaction rate should have decreased. Notably, at the same time, the yield of GPR drastically decreased. The yield of GPR should depend on the degree of dethreading. The dethreading could be prevented by the grafting reaction from the ends of the backbone. Thus, at low temperatures, the growth of PCL from the ends was slower than the dethreading. The high molecular weight of GPR indicated that the grafting polymerization proceeded slowly but steadily, once the end groups grew enough to prevent the dethreading. As mentioned above, dethreading is initiated with a basic catalyst, because the base prevents hydrogen bond formation between the α-CDs. Meanwhile, prevention of the hydrogen bond formation is necessary for the α-CDs to access the monomers. Therefore, the kinetic control of both the dissociation of the hydrogen bonds and the polymerization is necessary.

A stepwise increase in the reaction temperature yielded GPRs with relatively long graft chains in good yields (run 2). First, the reaction mixture was stirred at room temperature, so that the columnar crystals of the α-CDs in the pPR dissociated. Since the dissociation can induce dethreading, the reaction temperature was subsequently increased to accelerate the polymerization. Increased temperatures obviously improved the yield of GPR. Although the yield was decreased more than that of run 1 at a high temperature, the obtained GPR had a molecular weight that was twice as large. The ^1^H NMR of the GPR (Figure 3b) indicated that the increase in the molecular weight was due to the degree of polymerization of PCL. From the spectrum, the average molecular weight of PCL attached to a single α-CD was estimated to be ca. 9900. This molecular weight of the graft chain was similar to the one previously synthesized from a hydroxypropylated polyrotaxane by conventional methods composed of the five aforementioned reaction steps [10]. The coverage was very roughly estimated to be 25%, although the signals attributed to the α-CD were much smaller than those of others. The reduced coverage compared to that obtained in run 1 may suggest enhanced dethreading owing to the slower growth from the polymer ends at low temperatures, which was consistent with the reduced yield of GPR. Owing to the long or numerous graft chains, the obtained GPR could be dissolved in relatively nonpolar solvents such as toluene and chloroform, similar to a previously reported GPR, whereas the solubility of the GPR with short or sparse chains obtained in run 1 was not significantly different from that of PR without graft chains. Toward that end, we successfully simplified the synthesis of GPR.

## Conclusion

We demonstrated a novel synthetic method toward GPRs consisting of a PEG backbone and α-CDs modified with PCL graft chains. The GPRs were readily obtained by a three-step reaction, which included the activation of the end groups of PEG, complex formation of the PEG derivative and α-CD, and simultaneous end-capping/grafting reactions. The PCL chains simultaneously grew from the rings and backbone ends of the pPR. Although the PCL chain was not bulky enough to prevent the dethreading, the star-like polymer PCL at the backbone ends served as a bulky capping group. This end-capping process drastically simplified the synthesis of the GPR. Furthermore, the length of the graft chains and the yield of GPR depended on the kinetics of propagation from the CDs and backbone ends and the dethreading initiated by the dissociation of the hydrogen bonds between CDs. Since this synthetic scheme is, in principle, applicable to a variety of GPRs, this method should progress the research regarding the material properties of various polyrotaxanes with different graft chains.

## Experimental

### Materials

3-Amino-*n*-propyl-terminated PEG (PEG-amine) was purchased from Nichiyu Co., and had *M*_n_ and *M*_w_ of 18,400 and 21,200, respectively, as determined by size-exclusion chromatography (SEC) with a calibration curve using PEG standards. The standard polymers for the calibration of the molecular weights by SEC were purchased from Polymer Source Inc. α-Cyclodextrin (α*-*CD) was purchased from Nihon Shokuhin Kako Co. Ltd. 1,8-Diazabicyclo[5.4.0]undec-7-ene (DBU) was purchased from Aldrich. ε-Caprolactone and all other chemicals were purchased from Wako Pure Chemical Industries, Ltd., and all reagents were used as received without further purification.

### Measurement

^1^H NMR spectra (400 MHz) were recorded on a JEOL JNM-AL400 spectrometer at 298 or 343 K. The chemical shifts were calibrated using CHCl_3_ (7.26 ppm) or DMSO (2.50 ppm) as internal standards. Attenuated total reflectance-Fourier transform infrared (ATR-FTIR) spectra were recorded on Nicolet 4700 (Thermo Electron Co., Ltd.) equipped with a diamond attenuated total reflection (ATR) accessory (DurasamplIR II, SensIR Technologies Technologies) in air. SEC was performed on TOSOH HLC-8220 with two TSKgel Super AWM-H columns, with DMSO at 50 °C in the presence of 0.01 M lithium bromide as the eluent using RI detection and PEG standards. The flow rate was 0.5 mL/min.

### *N*-(3-PEG-*n*-propyl)gluconamide (PEG-GA)

3-Amino-*n*-propyl-terminated PEG (PEG-amine) (1.00 g) and δ-gluconolactone (356 mg) were dissolved in methanol (20 mL), and the resulting solution was refluxed overnight. The solution was cooled to room temperature and was subsequently concentrated. Dichloromethane was added to the viscous solution, forming a turbid solution. The solution was washed with a saturated saline solution repeatedly, and the organic phase was collected. The solution was evaporated and dried under vacuum to obtain PEG-GA (900 mg) as a white solid. ^1^H NMR (400 MHz, CDCl_3_, 298 K) 7.59 (amide), 4.71 (H2 of GA), 4.28 (H3 of GA), 4.08 (OH of GA), 3.98 (H6_a_ of GA), 3.64 (PEG), 2.97 (OH of GA), 1.82 (β-methylene of amide); IR (cm^−1^): 2884 s, 1649 m, 1537 w, 1467 m, 1360 m, 1344 s, 1281 s, 1242 m, 1148 s, 1113 s, 964 s, 844 s; SEC (PEG standards, DMSO/LiBr eluent): *M*_n_ = 20,600, *M*_w_ = 23,700, *M*_w_/*M*_n_ = 1.15.

### Pseudo-polyrotaxane

PEG-GA (300 mg) was dissolved in deionized water (2.4 mL). A saturated solution of α-CD in deionized water (1.20 g/7.5 mL) was added to the PEG-GA solution and then stirred at room temperature overnight. The solution became a white turbid gel. The gel was freeze-dried to obtain pPR as a white powder (1.50 g).

### Grafted polyrotaxanes (GPRs)

The pPR (100 mg) was mixed with ε-caprolactone (1.00 g) to obtain a suspension. DBU (100 mg) was added to initiate the polymerization, and then the reaction mixture was stirred at 0–130 °C for 7–48 h. The obtained mixtures were poured into methanol to precipitate the products, and then the precipitate was subsequently washed with methanol repeatedly. After drying under vacuum, GPRs were obtained as colorless solids. ^1^H NMR (400 MHz, DMSO-*d*_6_, 343 K) 5.7–5.4 (O2H, O3H of α*-*CD), 4.79 (C1H of α*-*CD), 4.5–4.3 (O6H of α*-*CD), 3.98 (C6H of PCL), 3.8–3.6 (C3H, C5H, C6H of α*-*CD), 3.51 (PEG), 3.4–3.2 (C2H, C4H of α*-*CD), 2.26 (C2H of PCL), 1.53 (C3H, C5H of PCL), 1.29 (C4H of PCL).
